# Inter‐Organ Crosstalk and Regulatory Mechanisms of Skeletal Muscle‐Derived Extracellular Vesicles in Systemic Metabolic Homeostasis

**DOI:** 10.1111/jcmm.70896

**Published:** 2025-11-05

**Authors:** Zhuoying Wu, Yuanyuan Gao, Qi Chen, Li Yuan

**Affiliations:** ^1^ Department of Endocrinology Union Hospital, Tongji Medical College, Huazhong University of Science and Technology Wuhan China

**Keywords:** adipose tissue, extracellular vesicles, liver, pancreas, skeletal muscle

## Abstract

Extracellular vesicles (EVs), including exosomes, play a pivotal role in intercellular communication by facilitating the transfer of bioactive molecules between cells. These vesicles, which encompass a variety of subtypes such as exosomes, microvesicles and apoptotic bodies, carry functional proteins, mRNAs, miRNAs and other molecular cargo that influence various physiological processes. In particular, skeletal muscle‐derived EVs have recently emerged as a novel category of myokines, contributing to muscle homeostasis through paracrine signalling and exerting systemic endocrine effects on metabolic tissues, including the pancreas, adipose tissue and liver. This review systematically examines the regulatory mechanisms of skeletal muscle‐derived EVs, with particular focus on exosomes, in mediating inter‐organ crosstalk. Additionally, it examines the factors that influence the release of skeletal muscle‐derived EVs, particularly exosomes and their subsequent effects on metabolism.

## 1 Introduction

Skeletal muscle, the largest organ in mammals, plays a pivotal role in movement, respiration and systemic metabolic regulation [1]. Beyond its mechanical functions, skeletal muscle coordinates multi‐organ physiology through intricate intercellular communication networks [2]. This coordination relies on highly differentiated tissues interconnected via dynamic signalling systems, where diverse cell types (including myofibers, fibroblasts and immune cells) interact critically to maintain muscle homeostasis and functionality [3]. Over the past two decades, skeletal muscle has been redefined as a novel endocrine organ [4, 5], primarily due to its capacity to secrete hundreds of signalling molecules termed myokines [6]. These muscle‐derived cytokines and peptides, expressed and released by myofibers, exert autocrine, paracrine and endocrine effects to regulate local and systemic functions [7]. Seminal studies established the ‘myokine hypothesis’ through discoveries like the exercise‐induced secretion of interleukin‐6 (IL‐6) into circulation, mediating skeletal muscle–liver metabolic crosstalk [8].

It has become increasingly evident that, with a deeper understanding of skeletal muscle endocrinological functions, researchers have gradually recognised that skeletal muscle not only regulates physiological processes through myokines but also communicates intercellularly via EVs. EVs are heterogeneous, membrane‐bound structures secreted by cells, broadly classified into subtypes such as exosomes, microvesicles and apoptotic bodies based on their biogenesis pathways and physical properties [9, 10]. These vesicles play critical roles in intercellular material transfer and communication by transporting bioactive molecules, including proteins, lipids and nucleic acids, between cells [11]. EVs secretion by skeletal muscle significantly expands their endocrine repertoire, enabling multifaceted regulation of systemic metabolic homeostasis.

Among EVs, exosomes represent a distinct subtype characterised by a defined diameter (30–150 nm) and endosomal origin [12]. Their unique bilayer membrane architecture enables evasion of mononuclear phagocyte system clearance, thereby achieving long‐range targeted delivery to distant tissues [13]. Crucially, exosome‐mediated transfer of signalling molecules orchestrates essential intercellular communication, establishing these vesicles as pivotal mediators in inter‐organ crosstalk research [14, 15]. Emerging evidence underscores exosomes as a novel myokine class with escalating investigative focus in skeletal muscle endocrinology [16]. Beyond secreting soluble factors alone, skeletal muscle releases exosomes that encapsulate canonical myokines (e.g., IL‐15, FGF‐21) [8], microRNAs (miRNAs) [17, 18] and other regulatory molecules. These exosomes deliver their cargo to target cells via membrane fusion or endocytosis, inducing transcriptomic reprogramming [19]. Their functional superiority over soluble mediators derives from three intrinsic properties: (i) enhanced stability through vesicular encapsulation that safeguards cargo from enzymatic degradation [9, 20]; (ii) targeted delivery mediated by surface adhesion molecules enabling tissue‐specific tropism [20]; and (iii) multi‐modal signalling capacity via simultaneous transfer of proteins and gene regulatory RNAs [21]. Collectively, these properties consolidate exosomes as pivotal integrators within skeletal muscle's endocrine signalling network, driving their expanding role in systemic metabolic orchestration.

While myokines have been extensively characterised, skeletal muscle‐derived EVs represent an emerging class of endocrine mediators with complex relationships to canonical myokines signalling. Recent evidence has identified skeletal muscle‐derived EVs not merely as passive carriers, but as dynamic endocrine effectors that exhibit selective overlap with canonical myokines, while also possessing distinct biological properties. This overlap refers to the fact that certain myokines, such as FNDC5/Irisin [22], can be actively transported via skeletal muscle‐derived EVs, demonstrating functional overlap with canonical endocrine signalling. Another distinct biological property refers to the ability of skeletal muscle‐derived EVs, beyond serving as passive carriers, to deliver a variety of cargoes, including regulatory RNAs (miRNAs/lncRNAs), metabolites and immunomodulators, thereby enabling multidimensional intercellular communication not available through canonical myokines alone [16].

In summary, skeletal muscle functions not merely as a mechanical organ for locomotion, but as a pivotal endocrine regulator of systemic metabolic homeostasis. Through the secretion of myokines and diverse EVs, it orchestrates inter‐organ communication, thereby establishing sophisticated cross‐tissue signalling networks. Among EVs, exosomes have emerged as particularly compelling mediators due to their enhanced stability, intrinsic targeting capacity and ability to deliver multimodal signalling cargoes. While exosomal roles in skeletal muscle metabolism are increasingly delineated, other EV subtypes contribute significantly to systemic metabolic regulation. This review systematically examines the regulatory mechanisms of skeletal muscle‐derived EVs, with particular focus on exosomes, in mediating inter‐organ crosstalk, highlighting their therapeutic promise for metabolic disorders exemplified by type 2 diabetes mellitus (T2DM) and obesity‐associated hepatic steatosis, among others.

## 2 Overview of EVs

**FIGURE 1:**
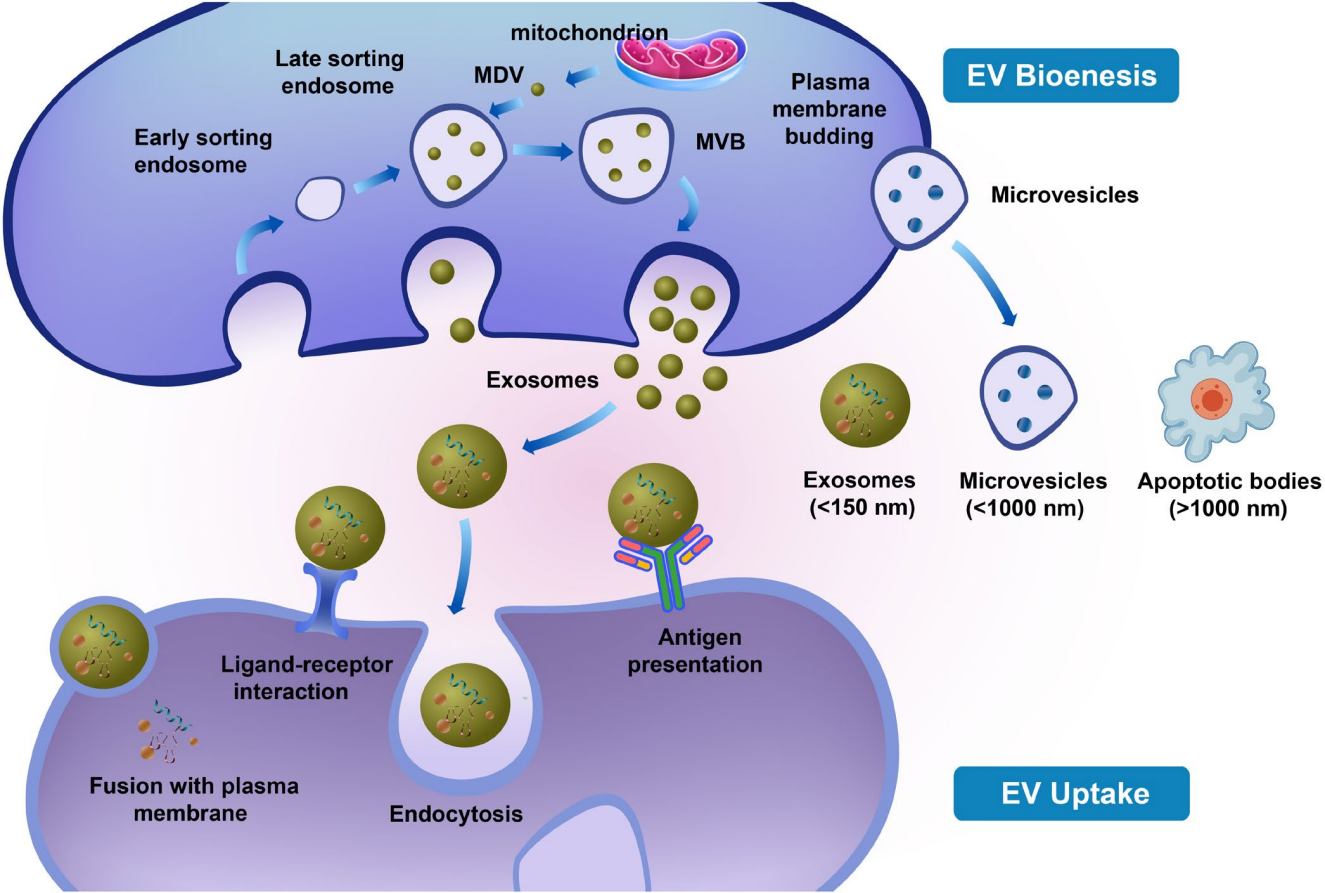
Biogenesis and uptake of EVs.

EVs are membrane‐bound particles shed from cells which carry their cargo of proteins, mRNAs and miRNAs, serving as key mediators of intercellular communication. They are broadly classified into subtypes such as exosomes, microvesicles and apoptotic bodies based on their biogenesis pathways and physical properties [23]. EVs interact with recipient cells by docking onto the target cell surface and facilitating the transfer of their cargo, which can modulate cellular differentiation and function [24]. Three subtypes of EVs have been extensively characterised: exosomes (30–150 nm), which are generated through the endosomal pathway and released upon multivesicular body fusion with the plasma membrane; microvesicles (< 1000 nm), which are produced by outward budding of the plasma membrane; and apoptotic bodies (> 1000 nm) [23, 24] (Figure 1).

Exosomes are complex vesicles composed of a diverse array of biomolecules, including specific proteins, lipids and nucleic acids of endosomal origin [25]. Their formation begins with the sequential invagination of the plasma membrane, leading to the creation of multivesicular bodies (MVBs). These MVBs intersect with other intracellular vesicles and organelles, ultimately determining the final composition of exosomes [26]. The maturation of exosomes involves two distinct pathways: the endosomal sorting complex required for transport (ESCRT)‐dependent pathway and the ESCRT‐independent pathway [27]. Once fully formed, exosomes are released into the extracellular matrix through fusion with the plasma membrane. They deliver their cargo to target cells via multiple mechanisms, including endocytosis, direct membrane fusion, receptor‐ligand interactions or binding to homologous receptors on the target cell membrane, which subsequently triggers intracellular signalling cascades [28]. Notably, approximately 70% of these functional interactions are mediated by miRNAs, while proteins account for only about 10% of the interactions [29].

Exosomes are generated through the inward budding of the late endosomal membrane, resulting in the formation of intraluminal vesicles inside multivesicular bodies (MVBs). These MVBs may fuse with the plasma membrane to release the internal vesicles, termed exosomes (30–150 nm), into the extracellular space. Additionally, mitochondrial‐derived vesicles (MDVs), which range from 70 to 150 nm in size, can merge with endosomes or MVBs and thereby contribute to the exosomal population. In contrast, microvesicles (< 1000 nm) are produced via the outward budding of the plasma membrane. EVs engage with recipient cells through mechanisms such as ligand–receptor interactions or antigen–antibody. The predominant uptake pathways include endocytosis and direct membrane fusion. Following internalisation or fusion, the cargo contained within EVs is delivered into the recipient cells [30].

## 3 Skeletal Muscle‐Derived Exosomes Mediate Communication Between Organs Through Paracrine or Endocrine Signalling

**FIGURE 2:**
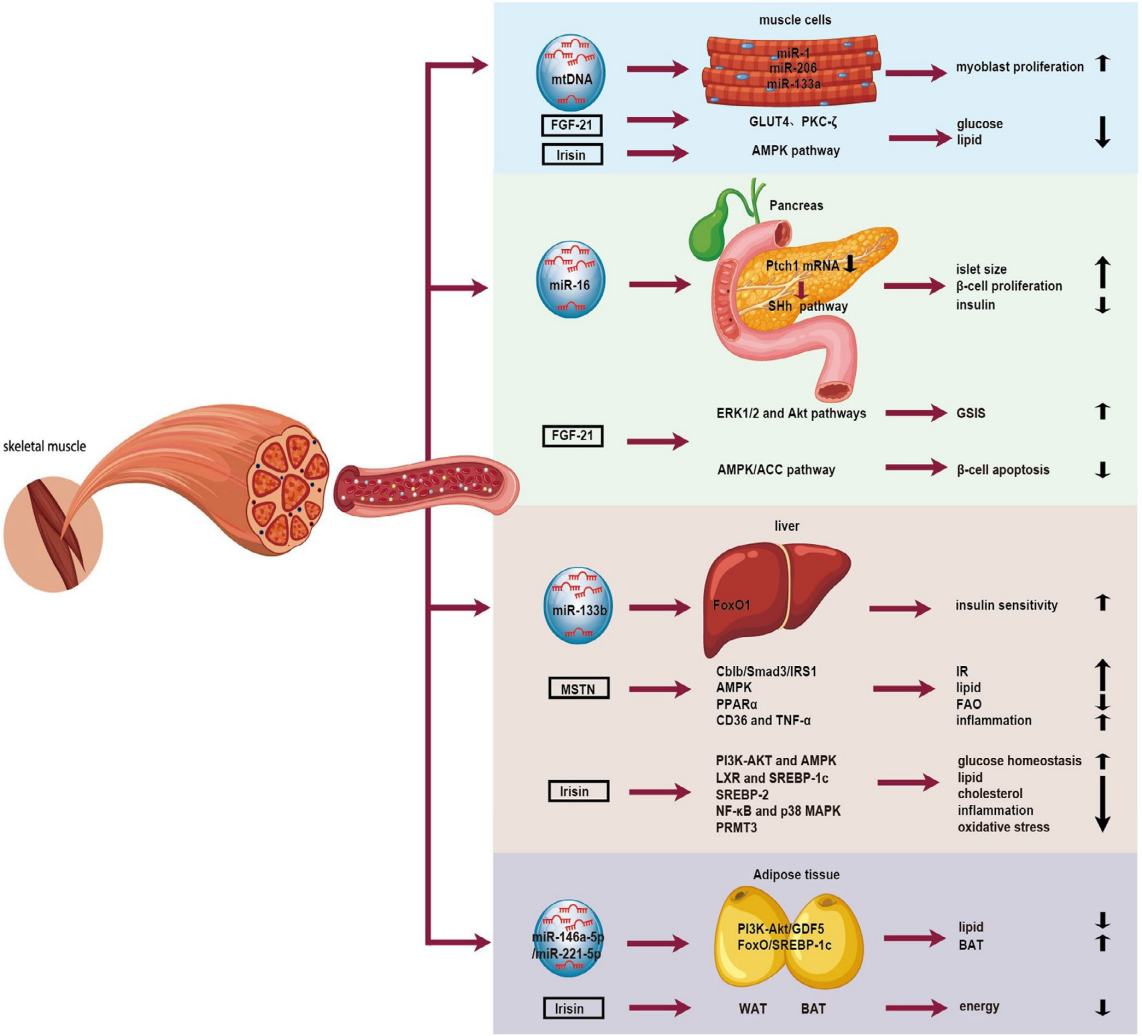
Mechanisms by which skeletal muscle‐derived exosomes regulate inter‐organ metabolic crosstalk via miRNA cargo in comparison with classical myokines.

Over the past two decades, skeletal muscle has garnered increasing research attention due to its emerging role as a multifunctional endocrine organ. Beyond the well‐characterised secretion of myokines, skeletal muscle also releases exosomes—nanoscale vesicles that encapsulate myokines and other bioactive molecules. These exosomes can be taken up by skeletal muscle itself or distant tissues, thereby mediating intricate communication networks both within skeletal muscle and between skeletal muscle and other organs (Figure 2) [31, 32].

This figure summarises how skeletal muscle‐derived exosomes modulate key metabolic processes, including insulin sensitivity, glucose and lipid metabolism and inflammatory responses, in target organs such as skeletal muscle (autocrine), pancreas, adipose tissue and liver through the delivery of specific miRNAs. For comparison, the diagram also includes soluble mediators representative of classical myokine signalling (e.g., Irisin, IL‐6).

### 3.1 Skeletal Muscle‐Derived Exosomes Mediate Crosstalk Within Skeletal Muscle Through Paracrine Signalling

A growing body of research indicates that exosomes play a pivotal role in transmitting intercellular signals between muscle cells and other cell types, leading to phenotypic changes in target cells [15, 33, 34]. For instance, Guescini et al. demonstrated that C2C12 mouse myoblast cells release exosomes containing mitochondrial DNA (mtDNA) and functionally relevant proteins. These exosomes facilitate the transfer of mtDNA into the mitochondria of target cells, thereby completing signal transduction [35]. Additionally, exosomes derived from the skeletal muscle of high‐fat diet (HFD)–treated mice have been shown to induce myoblast proliferation and modulate the expression of genes involved in muscle cell cycle regulation and differentiation. Comparative studies between myoblasts cultured in normal serum and those grown in exosome‐depleted serum revealed impaired myotube formation in the latter, underscoring the critical role of skeletal muscle‐derived exosomes in promoting myogenesis [36]. Furthermore, during myoblast proliferation, the levels of myokines such as miR‐1, miR‐206 and miR‐133a are significantly reduced [37]. These findings suggest that skeletal muscle may facilitate crosstalk between mature muscle fibres and myoblasts by secreting exosomes that carry specific biochemical signals essential for myogenesis. Moreover, lipidomics analyses have revealed that exosomes also mediate the transfer of lipids between muscle cells [18]. Collectively, these studies highlight that exosomes secreted by skeletal muscle function as paracrine‐like signalling molecules, orchestrating communication between muscle cells [38].

In addition to their role in promoting myogenesis, skeletal muscle‐derived exosomes may also participate in regulating skeletal muscle metabolism through the action of myokines. Fibroblast growth factor‐21 (FGF‐21), a well‐characterised myokine, plays a critical role in modulating glucose transport and lipid metabolism. G. Rosales‐Soto et al. [39] demonstrated that FGF‐21 enhances glucose uptake in skeletal muscle fibres via a glucose transporter protein 4 (GLUT4)‐dependent mechanism, which relies on the activation of atypical protein kinase C‐zeta (PKC‐ζ) in skeletal muscle. Another key myokine, irisin, has also been shown to regulate skeletal muscle metabolism. In vitro studies reveal that irisin significantly enhances oxidative metabolism in C2C12 muscle cells and upregulates the expression of GLUT4. Furthermore, irisin promotes glucose and lipid metabolism in human skeletal muscle through the phosphorylation of AMP‐activated protein kinase (AMPK) [7]. Additional research highlights that exosomes isolated from the skeletal muscle of HFD mice (HFD‐ELV) differ significantly from those of standard diet‐fed mice (SD‐ELV). HFD‐ELV are associated with higher body weight, glucose intolerance and insulin resistance (IR). miRNA profiling of HFD‐ELV revealed elevated levels of miR‐16, suggesting that skeletal muscle insulin resistance may be linked to the release of exosomes containing specific miRNAs, potentially involving the ceramide signalling pathway [40].

### 3.2 Skeletal Muscle‐Derived Exosomes Mediate Crosstalk Between Skeletal Muscle and Pancreas via Endocrine Signalling

The biodistribution of skeletal muscle‐derived exosomes, as demonstrated in recent studies, indicates that exosomes labelled with the lipophilic fluorescent dye DiR, when administered via tail vein injection in mice, are readily taken up by multiple tissues, including the lungs, liver, spleen, brain, heart, pancreas and gastrointestinal tract, with the liver exhibiting the highest accumulation [31]. These exosomes can also be observed within the pancreatic islets, although the islets are not the primary site of distribution [31]. However, due to current research limitations, it remains challenging to precisely quantify the distribution of exosomes within the pancreatic islets [41, 42].

Emerging research supports the concept of crosstalk between skeletal muscle and the pancreas [43]. Pancreatic β‐cells secrete insulin to facilitate glucose uptake in insulin‐sensitive tissues such as skeletal muscle, thereby maintaining glucose homeostasis and preventing hyperglycemia or hypoglycemia. Conversely, β‐cells can receive regulatory signals from other organs to modulate insulin secretion. For instance, during hypoglycemia, the brain enhances sympathetic nervous activity to suppress insulin secretion, restoring blood glucose levels to normal [44]. Given that skeletal muscle is a major site for insulin‐mediated glucose uptake, it is plausible that skeletal muscle also communicates with the pancreas to regulate insulin secretion [45]. This hypothesis has been experimentally validated, as exosomes derived from HFD‐ELV were shown to increase islet size in vitro and promote the proliferation of MIN6 β‐cells without affecting insulin secretion [40]. In vivo, injection of HFD‐ELV resulted in hyperinsulinemia in mice and specifically targeted pancreatic β‐cells, enhancing their proliferation by modulating gene expression related to islet proliferation and development [31]. This effect is likely mediated by the upregulation of miR‐16, which reduces Ptch1 mRNA levels and subsequently stimulates β‐cell proliferation via the Sonic Hedgehog (SHh) signalling pathway [40].

In a T2DM model, skeletal muscle exhibited significant alterations in the expression of miRNAs such as miR‐133a, miR‐206 and miR‐16 [45]. These miRNA perturbations influenced beta cell viability and function while also enhancing glucose uptake and systemic metabolic homeostasis, and these effects were facilitated by extracellular vesicles. These findings collectively suggest that exosomes may serve as key mediators of skeletal muscle–pancreas crosstalk through endocrine mechanisms.

### 3.3 Skeletal Muscle‐Derived Exosomes Mediate Crosstalk Between Skeletal Muscle and Adipose Tissue via Endocrine Signalling

Both muscle cells and adipocytes originate from mesodermal precursors [46], indicating a potential for specific interactions between skeletal muscle and adipose tissue [47]. Research suggests that the Wnt signalling pathway plays a pivotal role in mediating muscle–adipose crosstalk, with Wnt/β‐catenin signalling promoting muscle cell growth while simultaneously inhibiting intramuscular fat deposition [46]. Conversely, skeletal muscle can regulate adipose tissue metabolism through the secretion of bioactive factors. A key example is the myokine irisin, which is secreted by muscle tissue and has been shown to induce the browning of white adipose tissue, thereby enhancing energy expenditure [48]. Additionally, leptin has been demonstrated to directly stimulate fatty acid oxidation (FAO) in skeletal muscle via activation of the AMPK pathway [49].

Skeletal muscle and adipose tissue (AT) play critical roles in systemic metabolic regulation through the release of secretory factors, including cytokines and exosomes. These factors are closely implicated in the pathogenesis of metabolic disorders such as obesity and T2DM [19, 50]. Exosomes and exosomal miRNAs (exo‐miRNAs) have emerged as highly selective and specific mediators of crosstalk between adipose and muscle tissues. Through endocrine and paracrine mechanisms, these exo‐miRNAs influence processes such as tissue regeneration, glucose and fatty acid homeostasis, mitochondrial function in skeletal muscle, as well as adipogenesis, lipolysis and adipose tissue browning. MiRNA profiling studies have identified miR‐146a‐5p and miR‐221‐5p as key regulators associated with the MAPK, adipocyte cytokine, PI3K‐Akt, FoxO and insulin signalling pathways [51]. Activation of the FoxO pathway, for instance, inhibits adipogenesis by downregulating the expression of sterol regulatory element‐binding protein 1c (SREBP‐1c) and glucokinase [52], while activation of the PI3K‐Akt pathway promotes brown fat formation mediated by growth differentiation factor 5 (GDF5) [53]. These findings suggest that muscle‐derived exosomes may play a significant role in regulating adipose tissue metabolism. Current research highlights several novel exo‐miRNAs that mediate muscle‐fat crosstalk [19], and advances in miRNA sequencing (miRNA‐seq) technology are expected to further elucidate additional exo‐miRNAs involved in these complex interactions.

### 3.4 Skeletal Muscle‐Derived Exosomes Mediate Crosstalk Between Skeletal Muscle and Liver via Endocrine Signalling

Exosomes are increasingly recognised as key players in obesity‐related non‐alcoholic fatty liver disease (NAFLD) and non‐alcoholic steatohepatitis (NASH). They may act as mediators, transporting exo‐miRNAs or proteins to communicate with hepatic cells through endocrine mechanisms, thereby contributing to obesity‐associated fatty liver. Substantial evidence indicates that exosomes derived from adipocytes, macrophages, intestinal epithelial cells, gut microbiota and pancreatic β‐cells can serve as mediators in the development of NAFLD in obesity. However, direct evidence demonstrating that skeletal muscle‐derived exo‐miRNAs facilitate liver communication in the context of obesity remains limited. One study found that exercise induces the release of exosomes carrying miR‐133b from trained skeletal muscle, which modulates hepatic FoxO1 expression, ultimately enhancing insulin sensitivity [54]. Given that exercise also combats obesity, it is plausible that obesity may disrupt normal skeletal muscle–liver communication by suppressing the secretion of exo‐miR‐133b, thereby exacerbating hepatic insulin resistance in obesity.

Skeletal muscle exosomes can also transport myokines, which are increasingly implicated in energy homeostasis and the pathogenesis of obesity‐related NAFLD [55]. This suggests a direct crosstalk between skeletal muscle and the liver. Notable myokines include myostatin (MSTN) and irisin [5, 56]. The mechanisms by which MSTN influences liver metabolism are not fully understood, but several potential pathways have been proposed. MSTN may induce insulin resistance by degrading insulin receptor substrate 1 (IRS1) through the E3 ubiquitin ligase Cbl proto‐oncogene B (Cblb) in a Smad3‐dependent manner. It may also increase hepatic lipid accumulation by inhibiting AMPK signalling, reduce fatty acid β‐oxidation by attenuating peroxisome proliferator‐activated receptor alpha (PPARα) signalling [57], and promote hepatic inflammation by upregulating CD36 and tumour necrosis factor‐alpha (TNF‐α) [58]. In contrast, irisin has been shown to improve glucose homeostasis by activating the PI3K‐AKT and AMPK pathways, prevent palmitic acid‐induced lipid accumulation by inhibiting hepatic lipogenesis regulators such as liver X receptor (LXR) and SREBP‐1c [59], reduce cholesterol levels by suppressing SREBP‐2 [60], and mitigate inflammation and oxidative stress through the NF‐κB, p38 MAPK and PRMT3‐dependent pathways [61].

## 4 Factors Influencing the Release of Skeletal Muscle Exosomes and Their Impact on Metabolism

### 4.1 Exercise Promotes the Release of Skeletal Muscle‐Derived Exosomes and Affects Metabolism

Skeletal muscle is a highly plastic organ capable of adapting its phenotype and function in response to exercise, mechanical load and metabolic demands. Substantial evidence indicates that physical activity enhances skeletal muscle performance and promotes systemic metabolic health through the activation of multiple signalling pathways [62], whereas physical inactivity represents a well‐established risk factor for various metabolic disorders, including muscle atrophy and insulin resistance [63, 64].

**FIGURE 3:**
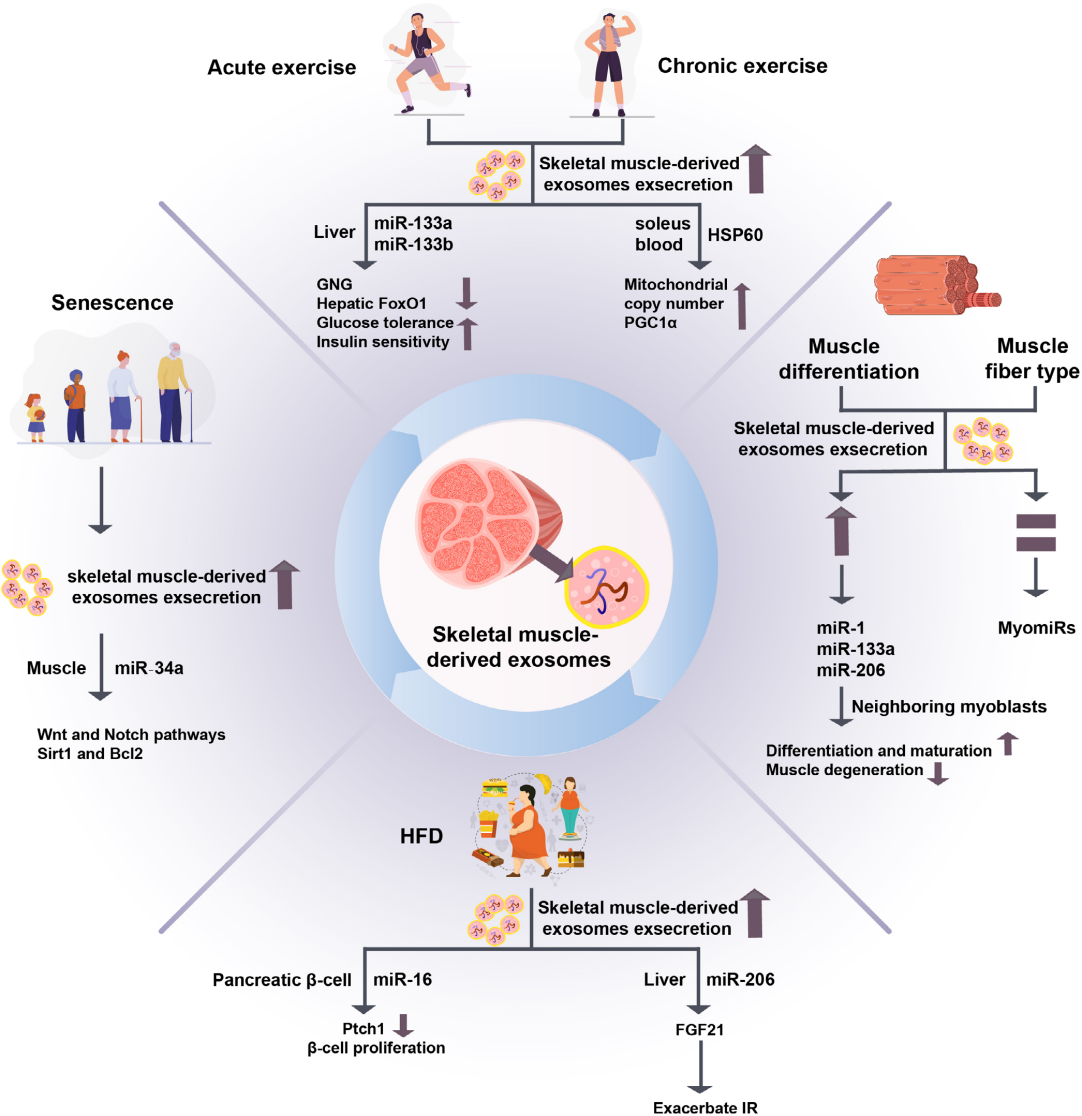
Diverse factors regulate the secretion of skeletal muscle‐derived exosomes and influence metabolic mechanisms in target organ.

Emerging research suggests that the metabolic benefits induced by exercise are partially mediated by skeletal muscle‐derived exosomes (Table 1) [68, 83, 84, 85, 86]. Exercise not only increases the quantity of skeletal muscle‐derived exosomes in circulation but also modifies their molecular composition [14]. Depending on the type of exercise (e.g., acute exercise vs. long‐term exercise), these exosomes carry temporally specific miRNA profiles that differentially regulate metabolic processes [87]. Acute exercise, high‐intensity interval training (HIIT), characterised by alternating periods of intense exercise and recovery, has been demonstrated to significantly increase maximal oxygen consumption (VO_2_max) [88], improve glycemic homeostasis [89] and enhance insulin sensitivity [90], thereby reducing the risk of metabolic diseases. This improvement is associated with the release of exosomes carrying specific microRNAs, which are secreted by muscle in response to exercise. Research has shown that exosomes from mice performing HIIT exhibit enriched levels of muscle‐specific miRNAs (myomiRs), such as miR‐133a and miR‐133b, which are associated with VO_2_max in humans, while showing reduced expression of obesity‐related miR‐192, compared to those from sedentary controls [54, 65]. These muscle‐derived exosomes contribute to systemic metabolic regulation by inhibiting hepatic FoxO1 expression, thereby suppressing gluconeogenesis, improving glucose tolerance and enhancing insulin sensitivity. Importantly, the administration of exosomes isolated from HIIT‐trained mice to sedentary counterparts resulted in improved glucose tolerance, insulin sensitivity and lipid metabolism [54, 66], underscoring the pivotal role of skeletal muscle‐derived exosomes in mediating the whole‐body benefits of exercise (Figure 3).

**TABLE 1:** Diverse factors regulate the secretion of skeletal muscle‐derived exosomes and influence metabolic mechanisms in target organ.

Factor	Exosome number	Enriched cargo	Organ target	Mechanism	References
Exercise					
Acute exercise	Increase	miR‐133a miR‐133b	Liver	Reduce GNG Inhibit hepatic FoxO1 Improve glucose tolerance Enhance insulin sensitivity	[54, 65, 66]
Long‐term exercise	Increase	HSP60	*soleus* Blood	Increases the mitochondrial copy number Increases PGC1α	[67, 68]
Senescence	Increase	miR‐34a	Muscle	Wnt and Notch pathways Sirt1 and Bcl2	[24, 69, 70, 71]
High fat diet	Increase	miR‐16	Pancreatic β‐cell	Decrease Ptch1 β‐cell proliferation	[31, 40, 68, 71, 72, 73]
		miR‐206	Liver	Exacerbate IR through FGF21	
Muscle					
Muscle fibre type	Similar	myomiRs	—	—	[34, 74]
Muscle differentiation	Increase	miR‐1 miR‐133a miR‐206	Neighbouring muscle cells	Favour the differentiation and maturation of neighbouring myoblasts Protect muscle from degeneration	[75, 76, 77, 78]
Neuronal innervation and activity	Increase	Neurotrophic microRNAs	Brain	Elevate neurotrophic myokines Elevate irisin and miRNAs	[79, 80, 81, 82]

Long‐term exercise facilitates the secretion of exosomes loaded with diverse molecular contents from skeletal muscle. Evidence indicates that aerobic exercise stimulates the release of exosomes of skeletal muscle origin into the bloodstream [68]. Mechanistic investigations have further demonstrated that chronic exercise promotes the secretion of muscle‐derived exosomes carrying Heat Shock Protein 60 (HSP60) and increases the mitochondrial copy number and the expression of three isoforms of peroxisome proliferator‐activated receptor gamma coactivator 1 alpha (PGC1α) [67]. These exosomes mediate inter‐tissue communication across key metabolic organs and contribute to the therapeutic potential of exercise in ameliorating metabolic disorders.

From a mechanistic perspective, exercise‐induced exosomes facilitate inter‐organ crosstalk via autocrine, paracrine and/or endocrine manners [91]. Through the delivery of myomiRs and other bioactive molecules, they modulate the expression of metabolism‐related genes in target tissues. Consequently, exosomes and their molecular constituents (e.g., the miR‐133 family) not only advance our understanding of exercise‐induced metabolic adaptations but also hold promise as novel therapeutic strategies and bioengineered agents for managing obesity, T2DM and age‐related metabolic disorders.

Various stimuli, including different forms of exercise, senescence, HFD, muscle fibre type composition and muscle differentiation status, can promote the release of skeletal muscle‐derived exosomes. These exosomes subsequently modulate metabolic processes in recipient tissues through the delivery of the corresponding cargo.

### 4.2 Senescence Promotes the Release of Skeletal Muscle‐Derived Exosomes and Affects Metabolism

Multiple studies have shown that senescent cells enhance their secretion of EVs relative to nonsenescent, healthy cells [70, 71]. Emerging evidence indicates that aging promotes enhanced secretion of exosomes from musculoskeletal tissues, particularly skeletal muscle [69]. Moreover, senescent cells have been shown to exhibit heightened secretion of EVs compared to their healthy counterparts [24], suggesting a broader phenomenon of altered vesicle release in aging. EVs derived from senescent musculoskeletal tissues encapsulate senescence‐associated miRNA (e.g., miR‐34a), which mediate paracrine senescence propagation to adjacent cells, thereby promoting tissue degeneration [24]. For example, the senescence‐associated miRNA miR‐34a targets Wnt and Notch pathways as well as cell survival factors Sirt1 and Bcl2 [69].

### 4.3 HFD Promotes the Release of Skeletal Muscle‐Derived Exosomes and Affects Metabolism

Current evidence indicates that HFD‐induced obesity upregulates exosomal secretion from skeletal muscle [73], which may be driven by metabolic stress and proinflammatory signalling cascades [71]. However, the molecular drivers of this process require rigorous investigation. Pathologically, HFD‐induced obesity is characterised by skeletal muscle insulin resistance [72], chronic low‐grade inflammation and mitochondrial oxidative stress, collectively forming a metabolic milieu that triggers exosomal release as a paracrine/endocrine stress response [68]. Consequently, HFD induces a significant increase in exosomes released by skeletal muscle in obese mice [40]. These exosomes contain molecules such as miR‐206, which exacerbates systemic insulin resistance by targeting hepatic FGF21 signalling [40]. This was confirmed in vitro by showing that increasing the amount of palmitate in the medium of C2C12 cells induced an increase in exosome release [31]. These results collectively confirm that high‐fat conditions induce enhanced secretion of exosomes from muscle cells.

### 4.4 Other Factors Influencing the Release or Function of Skeletal Muscle‐Derived Exosomes

Myogenesis is a highly regulated and intricate process, governed by myogenic regulatory factors (MRFs) [92]. MyomiRs, which are specifically expressed in muscle tissues, play a pivotal role in muscle function and development [93]. Among these, miR‐1, miR‐133a, miR‐133b and miR‐206 have been extensively studied for their roles in myogenesis [94, 95, 96, 97].

Previous research has demonstrated that hypoxic conditions can induce the release of EVs from various cell lines and tumours, with the characteristics of these EVs being influenced by the type of producing cells, as well as the duration and severity of hypoxia [98]. However, there is limited evidence to suggest that hypoxia affects the quantity or function of muscle‐derived EVs, as studies on the impact of hypoxia on skeletal muscle remain scarce [99]. Recent studies have successfully isolated EVs directly from five distinct skeletal muscle tissues of 6‐week‐old wild‐type male mice (extensor digitorum longus, soleus, tibialis anterior, gastrocnemius and quadriceps femoris) and incubated them under controlled conditions. These experiments revealed that muscle‐derived EVs were released even under potentially hypoxic conditions, which contrasts with the natural environment surrounding in vivo skeletal muscle. The findings indicated that the release of EVs from incubated muscle was not influenced by hypoxic conditions, and the release of myomiRs from skeletal muscle‐derived EVs was specific and independent of hypoxia [34].

Skeletal muscle tissues vary significantly in size, weight and fibre composition. For instance, the extensor digitorum longus and soleus are the smallest and lightest muscles, while the quadriceps femoris is the largest and heaviest. Additionally, the extensor digitorum longus, tibialis anterior, gastrocnemius and quadriceps femoris are predominantly composed of glycolytic Type IIB fibres, whereas the soleus is primarily composed of slow and fast oxidative Type I and IIA fibres [74]. The function of each skeletal muscle can also differ based on its anatomical position and interactions with surrounding tissues. The aforementioned study further investigated whether different skeletal muscle tissues influence the secretion, release and functionality of EVs. It was found that all five muscles secreted similar quantities of EVs during a 24‐h incubation in culture medium. However, the tibialis anterior, gastrocnemius and quadriceps femoris contained higher levels of myomiRs in their EVs compared to the smaller extensor digitorum longus and soleus muscles [34]. These results suggest that different muscle tissues secrete varying amounts of myomiRs, a phenomenon unrelated to tissue size but potentially influenced by muscle fibre type and the selective packaging of EV contents.

Emerging evidence from several studies in vitro reveals that the secretion of muscle‐derived EVs increases during muscle differentiation [77]. This has been demonstrated using mouse C2C12 cells [78] as well as primary human myoblasts [75]. The miR‐1, miR‐133a and miR‐206 carried by skeletal muscle EVs have also been found to exhibit increased expression during myoblast differentiation. They can presumably favour the differentiation and maturation of neighbouring myoblasts and protect the skeletal muscle from degeneration [76].

Muscle‐derived myokines and exosomes also play a critical role in maintaining brain homeostasis. Given that neuromuscular diseases and denervation‐related disabilities affect muscle metabolism, some studies hypothesise that neuronal innervation and activity may regulate the secretory activity of skeletal muscle. This hypothesis was tested using an engineered neuromuscular tissue model composed of skeletal muscle innervated by motor neurons [100, 101, 102]. Innervated muscle exhibited elevated mRNA expression of neurotrophic myokines, such as interleukin‐6, brain‐derived neurotrophic factor and FNDC5, as well as peroxisome proliferator‐activated receptor γ coactivator 1α (PGC‐1α), a key regulator of muscle metabolism [79, 80, 81, 82]. Upon glutamate stimulation, innervated muscle secreted higher levels of irisin and exosomes containing a greater diversity of neurotrophic microRNAs compared to non‐innervated muscle. These secreted biofactors enhanced branching, axonal transport and spontaneous network activity of primary hippocampal neurons in vitro [103].

## 5 Conclusions

Skeletal muscle‐derived EVs exhibit considerable diagnostic potential, which can be categorised into two main aspects. First, these EVs hold promise as muscle‐specific biomarker sources. They carry a rich repertoire of myomiRs, such as miR‐1, miR‐133a, miR‐206 and miR‐486, whose expression levels closely correlate with the physiological state of skeletal muscle [14, 104]. Consequently, skeletal muscle‐derived EVs represent strong candidates for non‐invasive, real‐time diagnostic markers capable of reflecting skeletal muscle health status. Second, skeletal muscle‐derived EVs demonstrate significant diagnostic value in muscle‐related disorders. Within the context of muscle injury and regeneration, emerging evidence indicates that the molecular cargo of these EVs (e.g., miR‐1, miR‐133 and miR‐206) presumably favours the differentiation and maturation of neighbouring myoblast [77]. These dynamic changes in EVs are hypothesised to mirror ongoing damage and repair processes, suggesting that skeletal muscle‐derived EVs could potentially serve as minimally invasive biomarkers for monitoring disease progression in congenital myopathies.

In metabolic diseases such as obesity and diabetes, insulin resistance is often accompanied by disruptions in muscle metabolism. During the development of T2DM, miRNAs in skeletal muscle, including miR‐133a, miR‐206 and miR‐16, are significantly altered, thereby influencing β‐cell survival and function [45]. Circulating miRNAs contained in exosomes derived from skeletal muscle cells mediate intercellular communication with peripheral tissues to improve glucose uptake and systemic metabolism [87]. Specific proteins and lipids within skeletal muscle‐derived exosomes also play critical roles in the initiation or progression of diabetes [31]. These findings suggest that miRNAs related to glucose metabolism (e.g., miR‐133) or lipid metabolites carried by skeletal muscle‐derived extracellular vesicles may serve as indirect diagnostic indicators of metabolic health.

EVs analysis faces a cascade of challenges and limitations hindering its progress in research. Current detection methods limit precise EV localisation within small structures like islets. Current isolation challenges include distinguishing free from EVs‐encapsulated molecules and separating soluble factors, as well as isolating specific subtypes of EVs, particularly those derived from a single tissue source. In the earlier part of the review, it is pointed out that the biodistribution of skeletal muscle‐derived exosomes, as demonstrated in recent studies, indicates that exosomes labelled with the lipophilic fluorescent dye DiR, when administered via tail vein injection in mice, are readily taken up by multiple tissues, including the lungs, liver, spleen, brain, heart, pancreas and intestine [31]. However, due to the complex composition, small size and short half‐life of exogenous exosomes, monitoring the in vivo traffic of those exosomes remains highly challenging [41]. In fact, as noted in recent reviews, there are only a limited number of instances where optical, nuclear and magnetic resonance imaging have been applied for in vivo exploration [105]. Various methods have been tested for in vivo tracking of EVs [105]. In the published literature on exosomes, most exosomes are labelled using lipophilic fluorescent dyes, the near‐infrared (NIR) dye DiR [105], which only exhibits intense fluorescence when inserted into a lipid membrane [106]. NIR dyes are ideal for in vivo applications because of their excellent tissue penetration and minimal autofluorescence [106]. Other methods include the use of radioisotopes, such as ^99m^Tc‐HMPAO [107] and [^111^indium] ([^111^In]) [108] or lentivirus‐mediated CD63‐GFP [109]. More advanced cell‐engineering techniques, such as encoding luminescent proteins (e.g., *Renilla* [110] or *Gaussia* luciferase [111]), have also been used to label exosomes. Notably, consistent with prior observations on muscle‐derived exosomes, which show predominant hepatic accumulation [31], multiple biodistribution studies of exogenous exosomes labelled with a single approach have reported significant retention in organs exhibiting efficient phagocytic activity and abundant blood flow, including the liver, spleen, kidneys and lungs [41, 106]. Consequently, the liver serves not only as a key reference organ for evaluating and comparing the efficiency of exosomes labelling and bioimaging technologies, but also underscores the physiological relevance of skeletal muscle‐derived exosomes in modulating hepatic metabolic processes, such as glucose homeostasis and lipid metabolism, highlighting the importance of investigating their mechanistic role in liver regulation.

However, due to the complexity of biodistribution and the limitations of current detection methodologies, precise measurement of intra‐tissue distribution, particularly within small and dispersed structures such as pancreatic islets, remains challenging [42]. The difficulties in quantifying exosomal accumulation in minute architectures like islets can be attributed to several factors. Firstly, islets comprise only 1%–2% of the total pancreatic volume and are diffusely distributed, complicating specific detection [112]. Secondly, conventional in vivo bioimaging systems exhibit low spatial resolution that hinders accurate discrimination between signals originating from islets versus those from surrounding exocrine tissue [113]. And thirdly, the actual abundance of exosomes within islets is likely low, potentially falling below the detection threshold of commonly used technologies. This notion is corroborated by empirical biodistribution data: following the injection of DiR‐labelled skeletal muscle‐derived exosomes in mice, the fluorescence signal detected in the entire pancreas accounted for only approximately 5% of that observed in the liver [106].

Current technological limitations present two major challenges in the isolation and characterisation of EVs: firstly, the inability to fully and accurately distinguish between free soluble factors and those encapsulated within EVs; and secondly, the lack of robust methods to isolate specific subtypes of EVs, particularly those derived from a single tissue source such as skeletal muscle. Firstly, common methods used in the isolation of EVs are differential ultracentrifugation, density gradient separation and size exclusion chromatography [114]. These methods separate EVs through different centrifugation speeds, density gradients and molecular size differences. Although these techniques can effectively isolate EVs from bodily fluids, the EVs obtained are typically a mixture derived from multiple tissues. While specific markers, such as surface proteins, can provide insights into their cellular origin, it is not possible to fully and accurately distinguish between free soluble factors and those encapsulated within EVs [114].

Secondly, following isolation, EVs are subsequently defined by a set of well‐established protein markers (e.g., CD9, CD63, CD81, etc.) [115]. However, these markers cannot be used to identify exosomes derived from specific tissue types. Furthermore, the most compelling evidence for the skeletal muscle origin of EVs often comes from cargo analysis. For example, the significant enrichment of well‐established muscle‐specific miRNAs (myomiRs, e.g., miR‐1, miR‐133a/b, miR‐206, miR‐486) in EVs isolated following exercise serves as a primary indicator of their skeletal muscle source [14, 104]. Even when employing myomiRs as characterisation tools, achieving absolute discrimination and purification of skeletal muscle‐derived EVs remains unattainable. One contributing factor is the relative specificity of these molecular markers; although myomiRs such as miR‐1, miR‐133a and miR‐206 are highly enriched in skeletal muscle, studies have detected their basal expression in other tissues such as smooth muscle [116]. Furthermore, technical limitations, such as insufficient detection sensitivity and inadequate specificity of current isolation methodologies, also impede precise separation and analysis. Therefore, isolating pure skeletal muscle‐derived EVs and specific exosome subtypes from mixed vesicle preparations remains challenging, which hinders the exploration of their precise functions and mechanisms.

Considering these limitations, experimental design should carefully account for specific research objectives to obtain high‐quality, purity or specific subclasses of EVs and establishing global standardised guidelines remains essential to improve the reliability and reproducibility of EVs‐based research.

## Author Contributions

**Zhuoying Wu:** conceptualization (lead), visualization (lead), writing – original draft (equal), writing – review and editing (equal). **Yuanyuan Gao:** writing – original draft (equal). **Qi Chen:** visualization (supporting). **Li Yuan:** conceptualization (supporting), writing – review and editing (supporting).

## Ethics Statement

The authors have nothing to report.

## Consent

The authors have nothing to report.

## Conflicts of Interest

The authors declare no conflicts of interest.

## Data Availability

Data sharing not applicable to this article as no datasets were generated or analysed during the current study.
